# Supplementary material for: Analysis of the clinical and radiological outcomes of percutaneous cervical nucleoplasty: A case–control study
Source: PLoS One. 2022 Dec 12;17(12):e0278883. doi: 10.1371/journal.pone.0278883 (PMC9744319; doi:10.1371/journal.pone.0278883)
Supplement: S1 Appendix — (PDF) [file pone.0278883.s001.pdf]

中國醫藥大學附設醫院
CHINA MEDICAL UNIVERSITY HOSPITAL
台中市北區育德路2號
2 Yude Road, Taichung, 40447, Taiwan (R.O.C.)
TEL : 886-4-22052121

中國醫藥大學暨附設醫院研究倫理委員會

Tel: 886-4-22052121 ext: 1924　Fax: 886-4-2207-1478　台中市北區育德路 2 號

臨床試驗/人體研究通過證明書

計畫名稱：脊椎手術治療之臨床成效的長期追蹤評估
計畫編號/本會編號： / CMUH110-REC2-113
計畫主持人：脊椎中心邱正廸主治醫師
執行機構：中國醫藥大學附設醫院
通過日期：2021 年 07 月 11 日
計畫有效日期：2022 年 07 月 10 日
計畫書：Version 01, Date: 2021.05.24
中文摘要：Version 01, Date: 2021.05.27
英文摘要：Version 01, Date: 2021.05.24
受試者同意書：Version 02, Date: 2021.07.06
持續審查頻次：每 12 個月一次

上述計畫已於 2021 年 07 月 11 日經中國醫藥大學暨附設醫院研究倫理委員會第二審查委員會簡易審查通過。本委員會的運作符合優良臨床試驗準則及國內相關法令。委員會決議此計畫之持續頻次如上述所示。請在持續審查必須進行前二個月向本會檢送完整之期中報告。

此計畫任何部分若經更改，必須在執行前重新提交本會審查及核准。此外，計畫主持人必須依時通報嚴重不良事件及涉及受試者或其他人風險的非預期問題。

主任委員 [signature]

中國醫藥大學暨附設醫院 研究倫理委員會 台中市北區育德路2號

中　華　民　國　一　一　〇　年　七　月　十　二　日

CMUH

The Committee is organized and operates in accordance with ICH6 GCP regulations and guideline.
本委員會組織與運作皆遵守 ICH6 GCP 規定

中國醫藥大學附設醫院
CHINA MEDICAL UNIVERSITY HOSPITAL
台中市北區育德路2號
2 Yude Road, Taichung, 40447, Taiwan (R.O.C.)
TEL : 886-4-22052121

Research Ethics Committee

China Medical University & Hospital, Taichung, Taiwan

Tel: 886-4-22052121 ext: 1924 Fax: 886-4-2207-1478

Clinical Trial/Human Research Approval

Date：Jul. 12, 2021

Protocol Title：Long term follow-up of clinical outcome evaluations of surgical interventions in the spine

Protocol No. / CMUH REC No.： / CMUH110-REC2-113

Name of Principal Investigator：Cheng-Di Chiu (Attending Physician, Spine center)

Name of Institution：China Medical University Hospital

Date of Approval：Jul. 11, 2021

Date of Expiration：Jul. 20, 2022

Protocol：Version 01, Date: 2021.05.24

Chinese Synopsis：Version 01, Date: 2021.05.27

English Synopsis：Version 01, Date: 2021.05.24

Informed Consent Form：Version 02, Date: 2021.07.06

Frequency of Continuing Review：once per every 12 months

This is to certify that the above referenced research project has been expedited approved by the Research Ethics Committee (REC) II of the China Medical University and Hospital on Jul. 11, 2021. The REC is organized under, and operates in accordance with, the Good Clinical Practices guidelines and the governmental laws and regulations. The frequency of continuing review for the research project determined by the REC is mentioned above. Please submit a completed progress report at least two months before the time at which continuing review must occur.

All the amendments to the research project should be re-submitted and approved by the REC BEFORE implementation. Also, the principal investigator is required to report all serious adverse events and unanticipated problems involving risks to the subjects or others on time.

CHINA MEDICAL UNIVERSITY & HOSPITAL
The Research Ethics Committee
No. 2 Yude Road, Taichung, Taiwan, R.O.C.

Martin M-T Fuh MD, DMSci.
Chairman, Research Ethics Committee II
China Medical University & Hospital

CMUH
