# Supplementary material for: Analysis of the clinical and radiological outcomes of percutaneous cervical nucleoplasty: A case–control study
Source: PLoS One. 2022 Dec 12;17(12):e0278883. doi: 10.1371/journal.pone.0278883 (PMC9744319; doi:10.1371/journal.pone.0278883)
Supplement: Attachment — Submitted filename: Response to Reviewers.docx [file pone.0278883.s002.docx]

Andrea Giannini, PhD

Academic Editor

*PLOS ONE*

Dr. Giannini:

We appreciate the opportunity to revise our manuscript entitled, “Analysis of the Clinical and Radiological Outcomes of Percutaneous Cervical Nucleoplasty: A Case–Control Study.” The manuscript ID is PONE-D-22-22166, authored by Chien-Hua Chen et al. Our responses to the comments from the editors and reviewers are included below.

We acknowledge the reviewers’ constructive comments and have addressed each comment in our point-by-point response. We have incorporated various corrections into the revised manuscript, according to the suggestions made by the reviewers and editors. A detailed list of our replies to the reviewers’ comments and the changes made in response to these comments is provided on the following pages of the “Authors’ Response to Editors and Reviewers.” Please feel free to contact us with any questions.

Your kind consideration of our manuscript is highly appreciated.

Sincerely yours,

**Authors’ Response to Editors and Reviewers**

**Academic editor’s points**

1. Please ensure that your manuscript meets PLOS ONE's style requirements, including those for file naming. The PLOS ONE style templates can be found at <https://journals.plos.org/plosone/s/file?id=wjVg/PLOSOne_formatting_sample_main_body.pdf>

<https://journals.plos.org/plosone/s/file?id=ba62/PLOSOne_formatting_sample_title_authors_affiliations.pdf>

Answer: The manuscript has been amended to meet PLOS ONE's style requirements.

2. During the internal evaluation of the documents provided. It is in our understanding that the participants were enrolled into each surgical group independent of research, at the physician's discretion. As such we would be grateful if you could report this within the mansucript text to avoid confusion with a possible clinical trial. Please also remove the TREND check list and IRB approved study protocols from the supporting material as this is not required for your article type.

Answer: Thank you. We have reported it in the manuscript text to avoid confusion **(Lines 104–105)**. The unnecessary documents have been removed from the supporting materials.

3. Thank you for stating the following in the Acknowledgments Section of your manuscript: "Dr. Cheng-Di Chiu was supported by China Medical University Hospital [DMR-111-097] for providing technical and financial support." We note that you have provided funding information that is not currently declared in your Funding Statement. However, funding information should not appear in the Acknowledgments section or other areas of your manuscript. We will only publish funding information present in the Funding Statement section of the online submission form. Please remove any funding-related text from the manuscript and let us know how you would like to update your Funding Statement. Currently, your Funding Statement reads as follows: "CDC was supported by China Medical University Hospital [DMR-111-097] for providing technical and financial support <https://www.cmuh.cmu.edu.tw/Home/CmuhIndex_EN>. The funders had no role in study design, data collection and analysis, decision to publish, or preparation of the manuscript." Please include your amended statements within your cover letter; we will change the online submission form on your behalf.

Answer: Thank you for this reminder. The current Funding Statement in the online submission is correct so there is no need to update it. We have removed any funding-related text from the manuscript.

4. Your ethics statement should only appear in the Methods section of your manuscript. If your ethics statement is written in any section besides the Methods, please move it to the Methods section and delete it from any other section. Please ensure that your ethics statement is included in your manuscript, as the ethics statement entered into the online submission form will not be published alongside your manuscript.

Answer: Thank you. The ethics statement is now only in the Methods section in the revised version of the manuscript.

5. Please include captions for your Supporting Information files at the end of your manuscript, and update any in-text citations to match accordingly. Please see our Supporting Information guidelines for more information: <http://journals.plos.org/plosone/s/supporting-information>.

Answer: Thank you. We have added a caption and an in-text citation for the supporting information **(Lines 92 and 408)**.

6. Additional Editor Comments: Dear authors, the topic of the present article titled “Analysis of the Clinical and Radiological Outcomes of Percutaneous Cervical Nucleoplasty: A Case–Control Study” is very interesting, the paper and the aim falls within the scope of the journal but the article needs major improvements. Review some spellings. Methods and discussion should be modified and improved. I suggest improving the manuscript with the reviewers' comments.

Answer: Thank you. We have further improved the manuscript based on your suggestions and the reviewers’ comments.

**Reviewers’ points**

**Reviewer #1**

1. The study deals with the outcomes of symptomatic contained CHIVD treated with PCN or CT. The present study was designed as a case–control comparative study. Patients who indicated for PCN after a failed CT for more than 6 months were recruited. Could you please comment to these more recent papers and compare with your results? 2022 May 17;15:1433-1441. doi: 10.2147/JPR.S359512. eCollection 2022. Long-Term Clinical Results of Percutaneous Cervical Nucleoplasty for Cervical Radicular Pain: A Retrospective Cohort Study Judith Divera de Rooij 2020 Nov;23(6):553-564. The Effect of Percutaneous Nucleoplasty vs Anterior Discectomy in Patients with Cervical Radicular Pain due to a Single-Level Contained Soft-Disc Herniation: A Randomized Controlled Trial Judith de Rooij 2020 Jan;133:e205-e210. doi: 10.1016/j.wneu.2019.08.210. Epub 2019 Sep 5. Long-Term Clinical Outcomes of Percutaneous Cervical Nucleoplasty for Cervical Degenerative Diseases with Neck Pain and Cervical Vertigo Chungen Li 2019 May;22(3):E205-E214. Therapeutic Effects and Safety of Percutaneous Disc Decompression with Coblation Nucleoplasty in Cervical Vertigo: A Retrospective Outcome Study with 74 Consecutive Patients and Minimum 1-Year Follow-Up Shangfu Li

Answer: Thank you for the comments and suggestion. We have updated the manuscript, commented on these more recent papers, and compared their results with ours **(Lines 231–233, 265–270, 283–291)**.

**Reviewer #2**

1. The manuscript technically sound,the data support the data and conclusion ,the statistical analysis been performed appropriately and rigorously The manuscript presented in an intelligible fashion and written in standard English.

Answer: Thank you for your evaluation and comments.

**Reviewer #3**

1. The manuscript was good written and can be accepted. English language is good. The results were good arranged after well described materials and methods. The results were supported by figures. Discussion was good including conclusions.

Answer: Thank you for your evaluation and comments.

**Reviewer #4**

1. First of all, I apologize you to say rude things. This manuscript seems a kind of supplementary examination. Many previous manuscripts announced the effectiveness of PCN, nevertheless you tried to support it. Even the conclusion in this manuscript does not sound new and impressive. In line 78-79, you mentioned the chemical effectiveness of PCN using the literature written by Ren D et al. They had referred to the correlation between PCN and PLA2, not IL-1, IL-6 and TNF-alpha specifically. More precise information should be written in this manuscript.

Answer: Thank you for your comments. We have incorporated more precise information in the introduction, which addresses the impact of PCN on IL-1and IL-8 expression in disc tissue **(Lines 68–73)**. In addition to its degradation of PLA2 activity, another study provided evidence that nucleoplasty decreased IL-1 expression and promoted IL-8 expression, implying the impacts of nucleoplasty on disc regeneration.

2. Compare to other literatures concerning about PCN, the follow-up period (six months) seems to be shorter in this study. Although the VAS score or other indicators drastically and rapidly decreased in PCN group, gradual but continuous decrease was shown even in CT group. Longer follow-up may lead the different conclusion.

Answer: Thank you for your comments. We agree with the reviewer’s perspective on the follow-up period. A longer follow-up period may provide additional information on the recovery of the CT group and recurrent pain after surgery. We discuss the insufficient length of the follow-up period in the section on limitations **(Lines 296–298)**. To our knowledge, two studies with control participants had a longer follow-up period compared to the present study (ref. 11 & 23). However, our study recruited patients who had received CT for 6 months prior to enrollment. This means that the control participants in our study had already received 12 months of CT by the 6^th^ month of the study period, at the time when PCN still showed a higher effect on pain relief than CT. We emphasize that timely PCN treatment may be a better choice for reducing pain compared to continued and lengthy CT.
